# Seed Quality and Seedling Growth After Applying Ecological Treatments to Crimson Clover Seeds

**DOI:** 10.3390/plants14060839

**Published:** 2025-03-07

**Authors:** Ratibor Štrbanović, Branimir Šimić, Mariana Stanišić, Dobrivoj Poštić, Nenad Trkulja, Violeta Oro, Rade Stanisavljević

**Affiliations:** 1 Department of Plant Diseases, Institute for Plant Protection and Environment, 11040 Belgrade, Serbia; pdobrivoj@yahoo.com (D.P.); trkulja_nenad@yahoo.com (N.T.); viooro@yahoo.com (V.O.); 2 Agricultural Institute Osijek, 31000 Osijek, Croatia; branimir.simic@poljinos.hr; 3 Department of Plant Physiology, Institute for Biological Research “Siniša Stanković”—National Institute of the Republic of Serbia, University of Belgrade, 11000 Belgrade, Serbia; mariana.stanisic@ibiss.bg.ac.rs

**Keywords:** crimson clover, eco-friendly treatments, seed quality, seedling growth

## Abstract

The effect of different treatments on the seed quality of crimson clover (*Trifolium incarnatum* L.) from six localities in eastern Serbia was investigated. The aim of this study was to improve seed quality and seedling growth of *T. incarnatum* using eco-friendly treatments. Tests were carried out under laboratory and field conditions, using hot water and air-drying temperatures. Seed quality parameters included germinated seeds, dormant seeds, dead seeds, seedling growth, and abnormal seedlings, all expressed as percentages. The water absorption test confirmed the presence of physical seed dormancy in crimson clover. The best results were achieved with a 30-min hot water treatment, which increased water absorption and reduced the percentage of dormant seeds. Longer exposure times increased the percentage of dead seeds and abnormal seedlings, whereas shorter exposure times increased the percentage of dormant seeds and reduced germination. There was strong agreement between the germination results obtained under laboratory and field conditions. These findings illustrate new biotechnological approaches to enhancing seed quality.

## 1. Introduction

Crimson clover (*Trifolium incarnatum* L.) belongs to the family Fabaceae and the genus *Trifolium*, which includes approximately 300 species [1]. In Serbia, crimson clover is a component of meadow and pasture plant communities [2,3]. According to Aćić [4], crimson clover comprises 14% of the pastures in the *Molinio-Arrhenatheretea* and *Festuco-Brometea* classes in western Serbia, whereas, on Stol mountain in eastern Serbia, it accounts for less than 10% [5].

Natural pastures generally have an insufficient legume content and low forage production [6,7]. Pastures and natural meadows are located on soils with low pH and generally low fertility [8], a trend also observed in other parts of Europe [9,10,11]. Under these conditions, increasing the legume content is crucial not only for nitrogen fixation and forage production [12], but also for improving cultivation in organic agriculture [13]. Unlike many other forage legumes, crimson clover thrives in soils with pH values ranging from 5 to 8 and generally requires only modest soil fertility [14], a trait particularly characteristic of Eastern European pastures [15]. The plant can be used as green manure for vegetation restoration [16] and land bioremediation [17]. Crimson clover has proven effective in suppressing weeds in maize-growing areas when cultivated in mixtures with other plants [18] and performs well in crop rotations with vegetables [19,20,21,22]. It is particularly suitable as a pre-crop for nutrient supply in tomato cultivation [23] and as a cover crop in orchards and vineyards [24,25]. Crimson clover blooms very early in spring, producing brightly colored, decorative inflorescences that attract bees and other insects [26], making it a popular choice for flower strips [27].

The cultivation of crimson clover requires seeds of the highest quality. However, seeds from many plant families are known to exhibit periods of physical dormancy [28,29,30], a trait also evident in the Fabaceae family [31,32,33] and *Trifolium* species [34]. To reduce the physical dormancy of seeds, physical, chemical, or biological treatments can be applied to regulate slow seed water uptake. During this process, various genetic, physiological, and biochemical changes occur [35]. Seed dormancy is undesirable in agriculture as it inevitably reduces seed germination. Poor seed quality results in reduced germination and weak seedling growth, necessitating the use of larger amounts of seeds and reducing the plant’s competitiveness against weeds and other undesirable plants. This, in turn, increases production costs [36,37]. A high germination rate also depends on the proportion of dormant seeds, as there is a negative correlation between germinated and dormant seeds [38,39]. Various studies have used inorganic acids and other toxic chemicals [40] to soften the seed coat and make it more permeable (impact on physical dormancy), whereas the use of water and dry air is harmless to the human population and environment. However, the effectiveness of hot water in breaking seed dormancy and increasing germination varies from species to species [41,42].

This study aimed to break seed dormancy and improve seed quality and seedling growth in crimson clover using ecologically acceptable treatments.

## 2. Results

### 2.1. Weight of 1000 Seeds

The analysis of variance for 1000-seed weight, considering lot, year, and their interaction, did not reveal statistically significant effects (*p* ≥ 0.05), Table 1.

### 2.2. Water Absorption Test and the Effect of Treatments

The analysis of variance showed a highly significant (*p* ≤ 0.001) effect of the applied treatments on seed weight after water absorption. However, lot, year, and all interactions did not show a statistically significant effect (*p* ≥ 0.05) on weight gain after water absorption. Below are the means for lots and years.

The difference in the weight of 1000 seeds before applying the treatments ranged between 0.225 and 0.233 g and was not statistically significant (*p* ≥ 0.05) (Table 2).

**Figure 1:**
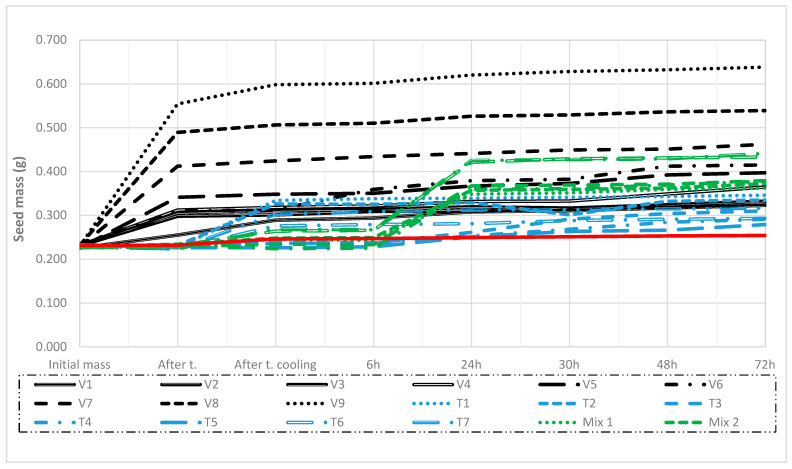
Effect of treatments on the seed weight of crimson clover after the water absorption test, carried out over three years on the means of six lots (Tukey’s multiple range test, *p* ≤ 0.05).

After the application of hot water treatments, the maximum weight increased by 126% for treatment V9 compared to the initial seed weight, and this increase was statistically significant (*p* ≤ 0.05). In the incubator, the seed weight for treatment V9 increased by 162% after 6 h, 171% after 24 h, 178% after 30 h, 176% after 48 h, and 179% after 72 h, all compared to the initial seed weight, with all increases being statistically significant (*p* ≤ 0.05). For treatment V8, the maximum seed weight increased by 111% after hot water treatment, but this increase was not statistically significant (*p* ≤ 0.05). However, after 72 h of storage in the incubator, the maximum seed weight increased by 133% compared to the initial weight, and this increase was statistically significant (*p* ≤ 0.05). Water intake and weight gain were minimal after air treatments T1–T7. After combined temperature treatments (MIX 6 and MIX 5), water absorption and weight gain increased by 87% and 82%, respectively, after 24 h in the incubator, and by 94% and 87%, respectively, after 72 h in the incubator, compared to the initial seed weight. These increases were not statistically significant (Figure 1).

#### 2.2.1. Seed Quality and Seedling Growth

The analysis of variance showed that the effect of treatments on all seed quality traits tested was statistically significant under laboratory conditions (*p* ≤ 0.05 to ≤ 0.001). In contrast, the factors lot and year, along with all interactions, had no significant effect on any of the seed quality traits tested (*p* ≥ 0.05) (Table 3).

**Figure 2:**
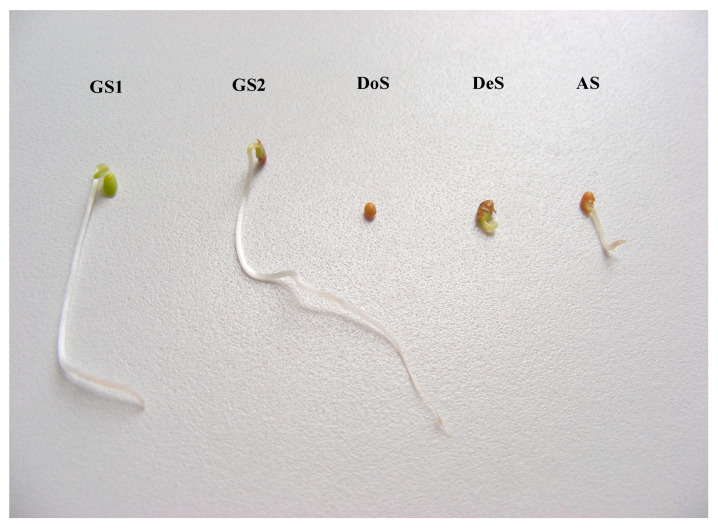
Seed quality in laboratory tests (GS1 first count, GS2 final count, DoS dormant seeds, DeS dead seeds, AS abnormal seedling).

**Figure 3:**
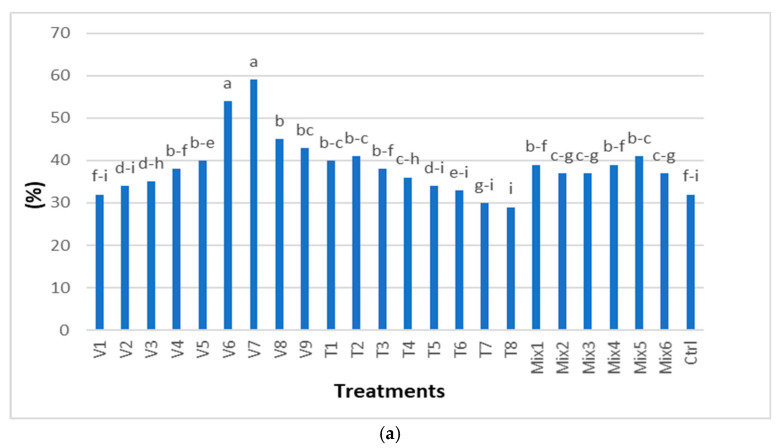

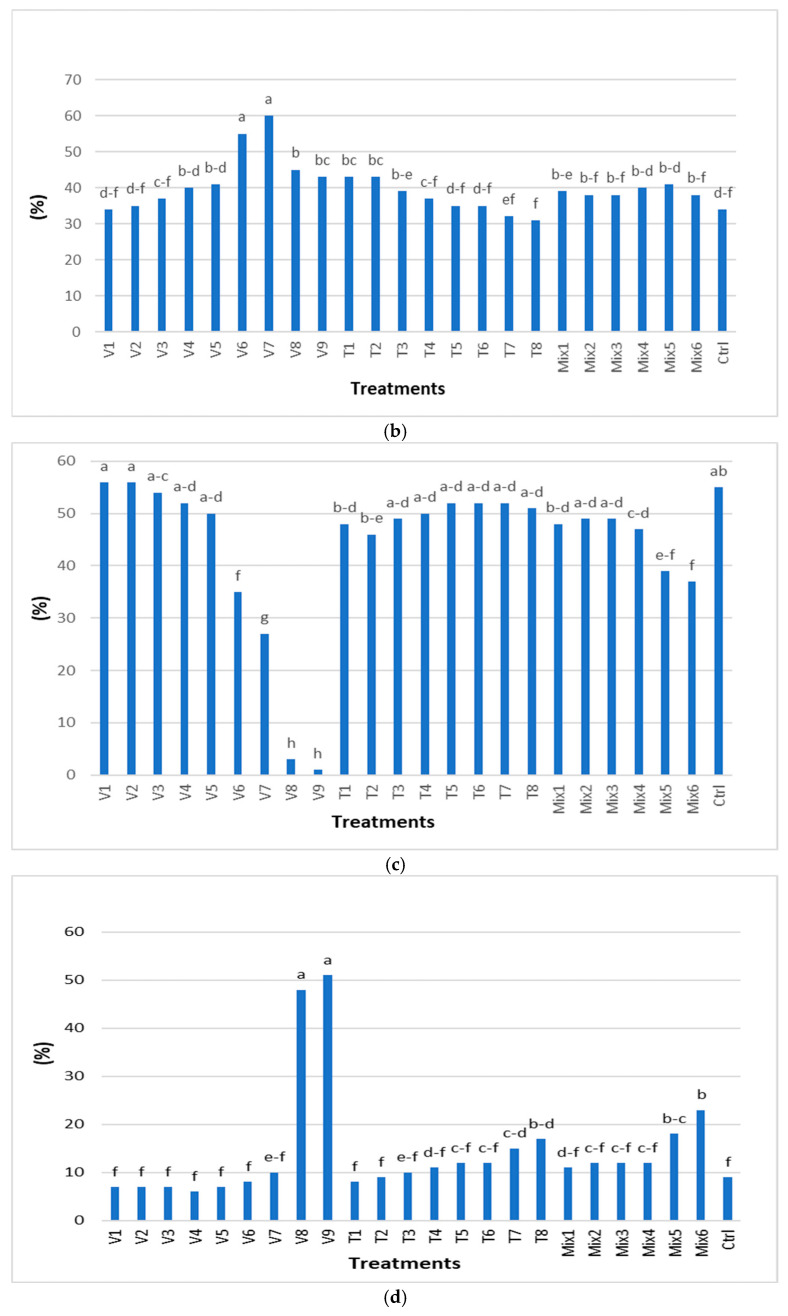

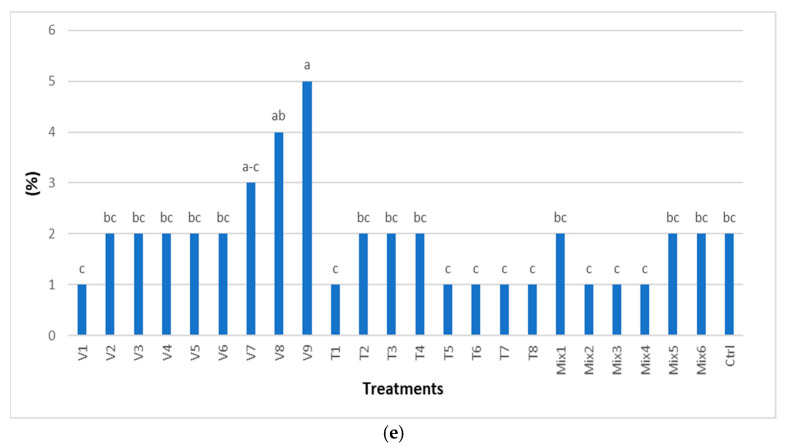
Seed quality after applied treatments (in %): (**a**) first germination count, (**b**) final germination count, (**c**) dormant seeds, (**d**) dead seeds, (**e**) abnormal seedlings. Tukey’s multiple range test, numbers followed by different letters are statistically different at *p* ≤ 0.05. Treatments: V1, Water 100 °C 2 min; V2, Water 100 °C 4 min; V3, Water 100 °C 6 min; V4, Water 100 °C 8 min; V5, Water 100 °C 10 min; V6, Water 100 °C 20 min; V7, Water 100 °C 30 min; V8, Water 100 °C 45 min; V9, Water 100 °C 60 min; T1, 40 °C 5 min; T2, 40 °C 10 min; T3, 60 °C 5 min; T4, 60 °C 10 min; T5, 80 °C 5 min; T6, 80 °C 10 min; T7, 100 °C 5 min; T8, 100 °C 10 min; Mix1, T40 °C 5 min + T-20 °C 10 min; Mix2, T40 °C 10 min + T-20 °C 10 min; Mix3, T60 °C 5 min + T-20 °C 10 min; Mix4, T60 °C 10 min + T-20 °C 10 min; Mix5, T80 °C 20 min + T-20 °C 20 min; Mix6, T100 °C 30 min + T-20 °C 20 min; Ctrl, Control treatment.

First germination count (GS1). Among the water treatment groups, treatments V7 and V6 were the most effective, with a 27% and 22% increase in germination, respectively, and were statistically significant compared to the control treatment. Among the air temperature groups, treatment T2 was the most effective, with a 9% increase in germination, which was statistically significant compared to the control treatment. Among the mixed temperature treatments, Mix 5 had a 9% increase in germination, compared to the control treatment and was statistically significant. Treatments V1, V2, T6-T8 were at the level of the control and were not statistically significant (Figure 2 and Figure 3a).

Final germination count (GS2). The optimal seed treatments for increasing germination included soaking the seeds in hot water (100 °C) for 30 min (V6) and 20 min (V7), which resulted in 26% and 21% higher germination, respectively, and were statistically significant compared to the control. Meanwhile, treatments with hot water for the two shortest exposure times (V1 and V2) did not significantly affect germination (Figure 2 and Figure 3b). Treatments combining high and low temperatures (Mix) had a fairly uniform and statistically significant effect, increasing the germination rate from 4% (Mix 6, 2, 3) to 7% (Mix 5) compared to the control (Figure 3). Germination rate at air temperatures of 40 °C (T1 and T2) resulted in a 10% increase, compared to the control, and the treatment was statistically significant. In contrast, germination rates at 100 °C (T7 and T8) increased by 2% and 3%, respectively, compared to the control, but these treatments were not statistically significant.

Dormant seeds. Depending on the water treatment duration, the percentage of dormant seeds varied from 56% (V1) to just 1% (V9). There were no significant differences between the least effective water treatments (V1 and V2, 56%, V3 54%, V4 52%) and the control (55%). For air temperature treatments, the percentage of dormant seeds at 80 °C and 100 °C (T5, T6, and T7) was 52%, and these treatments were statistically significant compared to the control. In contrast, at the air temperature of 40 °C, the percentage of dormant seeds (T2) was 46%, but this treatment was not statistically significant compared to the control (Figure 2 and Figure 3c).

Dead seeds. Seed mortality was the highest (51% and 48%) after water treatments with the longest exposure periods (V9 and V8). In contrast, treatments with shorter exposure periods (V5–V1) resulted in dead seed percentages of 7% and 5% compared to 4% in the control, with no significant differences among them. After air treatments T1–T8, the percentages of dead seeds (8–17%) were statistically higher in all treatments compared to the control treatment. Furthermore, all mixed treatments generated significantly more dead seeds, with 23% in Mix 6 and 11% in Mix 1, compared to the control treatment (Figure 2 and Figure 3d).

**Figure 4:**
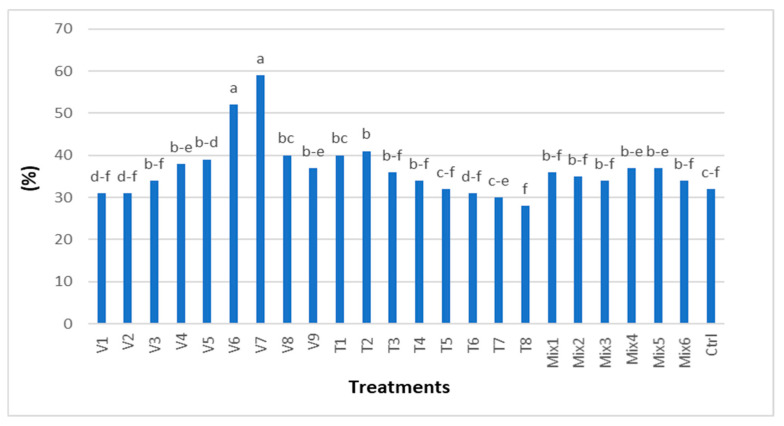
Seed germination (%) after applied treatments in field conditions. Tukey’s multiple range test, numbers followed by different letters are statistically different at *p* ≤ 0.05.

Abnormal seedlings. Among the quality traits analyzed, the percentage of abnormal seedlings varied the least (Figure 4). However, after the hot water treatment (V9) there were 3% more abnormal seedlings, which was statistically significant compared to the control. After V8 treatment, there were 2% more abnormal seedlings, which was not statistically significant compared to the control. All other treatments resulted in ±1% difference in abnormal seedlings compared to the control (Figure 2 and Figure 3e).

Seedling stem and root growth after applied treatments

Treatments V7 and V6 resulted in significantly higher stem increase (3.25 and 3.18 cm) compared to other treatments, except for V8 (2.89 cm). Meanwhile, treatments V1 and V2 with stem growth of 2.21 cm and 2.29 cm, respectively, treatments T8 to T5 with stem growth of 2.18 to 2.32 cm, respectively, and Mix 6 treatment with stem growth of 2.26 cm were the least effective, showing no significant differences from the control treatment (stem 2.19 cm) (Table 4).

The highest root growth was observed in treatment V7 (2.34 cm), followed by water treatments V6 (2.31 cm) and V8 (2.08 cm), as well as temperature treatments T1 (2.07 cm), T2 (2.08 cm), and T3 (2.06 cm), with no statistically significant differences among them (*p* ≥ 0.05). The lowest root growth was in the control treatment (1.72 cm) (Table 4).

#### 2.2.2. Seed Germination After Applied Treatments in Field Conditions

The field germination analysis showed results similar to those observed under laboratory conditions (Figure 3b and Figure 4).

By sowing seeds in soil and counting seedlings after 10 days, the average germination rate in soil (37%) was 3% lower than that in the incubator (40%), considering all treatments tested (Figure 3b and Figure 4).

Under soil conditions, after the application of hot water treatments V7 (100 °C, 30 min) and V6 (100 °C, 45 min), the highest germination percentages were achieved at 27% and 20%, respectively. These results were significantly different compared to the control.

Among the air temperature treatment groups, the T2 treatment (40 °C, 10 min) resulted in the highest germination percentage (41%), which was significantly different from the control (32%).

In the mixed treatment groups, Mix 5 (80 °C, 20 min) achieved the highest germination percentage (37%), which was also significantly different from the control.

Germination under field conditions showed no statistical differences between the control (32%) and the following treatments: V1–V3, T4–T8, Mix 3, and Mix 6 (Figure 4).

Seedling stem and root growth after applied treatments

Under field conditions, stem growth after seed treatments V7 and V6 was 3.29 cm and 3.26 cm, respectively, which was significantly higher than in the other treatments, except for treatment V8 (2.95 cm).

The control treatment had the least stem growth (2.24 cm) and was not significantly different (*p* ≥ 0.05) from treatments V1–V5 (2.28–2.52 cm), T1–T8 (stem 2.56–2.24 cm), Mix 1–Mix 6 (stem 2.51–2.31 cm) (Table 5).

Root growth was highest after V7 treatment (2.39 cm) but was not significantly higher (*p* ≥ 0.05) than root growth after V6 (3.26 cm) and V8 treatments (2.13 cm).

The lowest root growth was in the control treatment (1.75 cm), which was not significantly different (*p* ≥ 0.05) from treatments V1–V5 (1.81 cm to 1.94 cm), T4 to T8 (1.96 cm to 1.81 cm), and all mixed treatments (1.92 cm to 1.79 cm) (Table 5).

### 2.3. Principal Component Analysis (PCA)

PCA was applied to crimson clover seeds, considering both laboratory tests (germinated, dormant, dead seeds, abnormal seedlings, and stem and root growth) and field tests (germinated seeds; stem and root growth).

**Figure 5:**
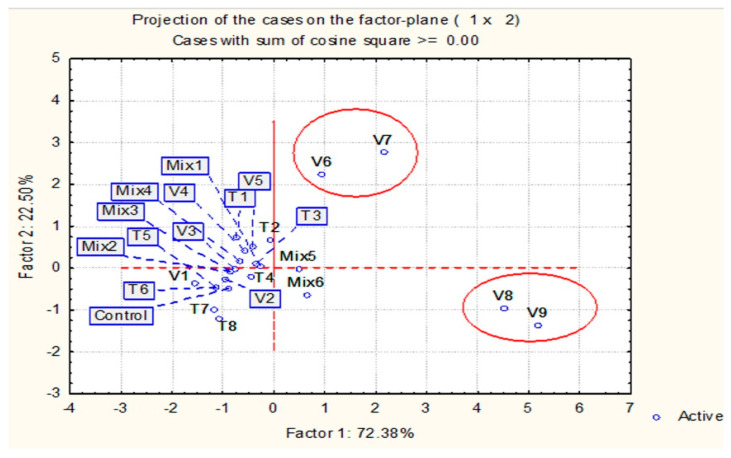
Principal component analysis (PCA) of treatment effects on seed germination, dormant seeds, dead seeds, and abnormal seedlings of crimson clover under laboratory conditions and germination test under field conditions.

The first and second principal components (PC-1 and PC-2) accounted for 72.38% and 22.50% of the total variance, and their mutual projections are shown in Figure 5. The analysis highlighted treatments V6 and V7 as part of the same group, which proved to be the most effective for germination.

The second group included treatments V8 and V9, which were the most effective in reducing dormant seeds. However, they also increased the number of dead seeds, which prevented them from effectively increasing germination.

Among all other treatments, including the control treatment, the PCA analysis showed a shorter distance (Figure 5).

## 3. Discussion

Regarding water absorption, the treatment in which seeds were exposed to hot water at 100 °C for 20 min was the most effective, resulting in the greatest weight gain and breaking seed dormancy (Figure 1). This indicates the presence of physical dormancy in crimson clover. According to Kelly et al. [43], physical seed dormancy is caused by a seed coat that is impermeable to water and/or gas. In the study by Galussi and Moya [44], the mechanism of physical dormancy in *Trifolium repens* seeds may be explained by a high concentration of hydrophobic components, such as polyphenols, lignin, condensed tannins, pectic substances, and a higher proportion of cellulose, which stiffen the cell walls. In our study, hot water treatments may have softened these components, facilitating water absorption and breaking seed dormancy.

The two longest water treatments (V9 and V8) did not achieve maximum germination due to a high percentage of dead seeds (51 and 48%, respectively) (Figure 3). On the other hand, water treatments with seed exposure times of up to 6 min (V1–V3) did not absorb enough water (Figure 1), resulting in a high percentage of dormant seeds (56–54%) and a low germination rate (Figure 3). Following air temperature treatments (T1–T8), the seed weight decreased, indicating a reduced water absorption rate. This was closely related to the high percentage of dormant seeds (Figure 3) and dead seeds, particularly in T7 and T8 (Figure 3). These treatments affected germination, ranging from negative (2% and 3% lower germination compared to the control treatment) to positive, with germination increasing by up to 10% compared to the control treatment (Figure 3). Mixed temperature treatments (Figure 1) increased the percentage of dormant seeds from 37% (Mix 6) to 49% (Mix 2 and 3) and increased the percentage of dead seeds from 11% (Mix 1) to 23% (Mix 6). Meanwhile, seed germination increased from 4% to 7% (Figure 3).

In our tests, the germination rate under laboratory conditions was 3% higher than under field conditions. This aligns with previous studies on pepper and tomato seeds [45] as well as rapeseed [46]. There are conflicting reports in the literature on the effect of different treatments on legume seed germination. According to Long et al. [47], seeds of *Astragalus* sp. were exposed to air temperatures from 60 to 80 °C for up to 48 h; however, only a small proportion of seed coats were permeable, resulting in limited dormancy breaking. Our findings regarding seed coat impermeability and low water absorption under similar conditions support these results (Figure 1 and Figure 3). For the seeds of *Astragalus* spp. (Fabaceae), mechanical and chemical (sulfuric acid) scarification treatments were found to be the most effective [48]. Similarly, seeds of *Bituminaria basaltica* and *B. bituminosa* (Fabaceae) showed that chemical, physical, and thermal scarification treatments were able to break physical seed dormancy, with mechanical and chemical scarification proving more effective than thermal scarification for both species [49]. For *Piscidia piscipula* (Fabaceae), the highest germination rates were recorded after hot water treatment, whereas mechanical scarification yielded worse results [50]. In *Trifolium pratense*, freezing seeds at −80 °C and immersing them in hot water at 90 °C increased germination rates from 26% to 74.5% [51].

According to Šerá [52], non-thermal plasma treatment of seeds can reduce seed coat hardness, which is associated with physical dormancy in many Fabaceae species, including *Trifolium*. This treatment has been shown to enhance seed germination and seedling growth [53].

According to Yan and Chen [54], seed dormancy and germination potential are affected by a variety of external and internal factors. However, the exact mechanisms beyond these phenomena are not completely understood.

Early plant establishment through seed germination and seedling emergence is a key process that determines seedling numbers, the distribution of emergence over time, and early seedling growth [55].

According to Ćupina et al. [56], achieving high annual forage yields in legume–grass mixtures can be obtained with proper selection of species and an appropriate legume–grass ratio. This is preceded by the successful establishment of grass–legume fodder mixtures, which require seeds of the highest quality and seedlings with strong initial growth [36]. All technologies used in agriculture must not negatively impact biodiversity, contribute to soil degradation, or harm the environment [57].

## 4. Materials and Methods

**Figure 6:**
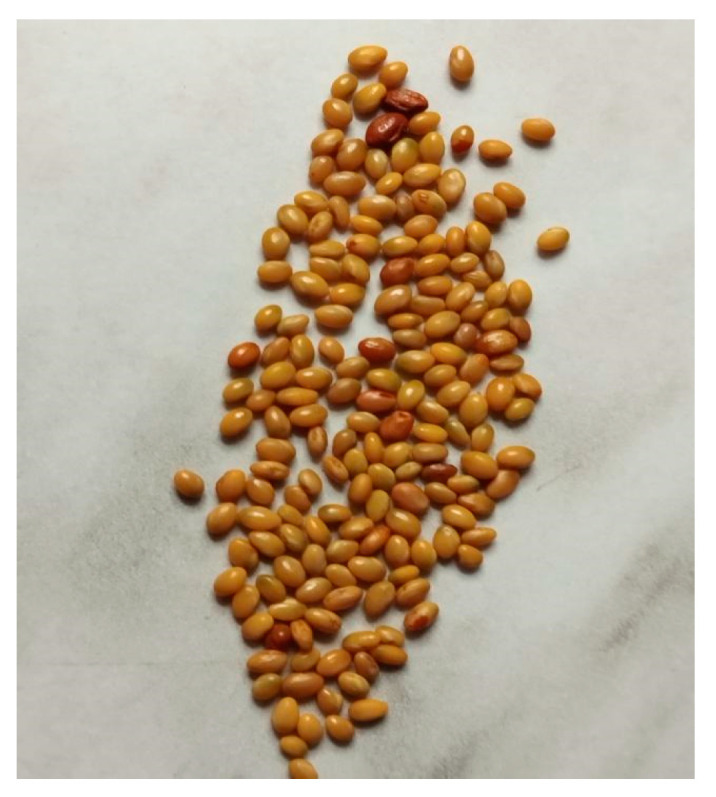
Crimson clover seeds.

**Figure 7:**
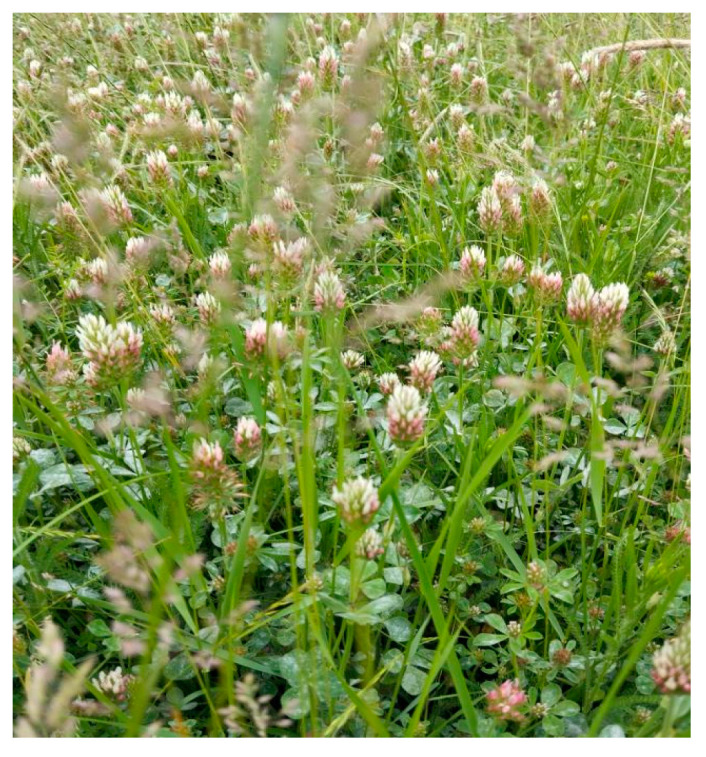
Crimson clover field.

**Figure 8:**
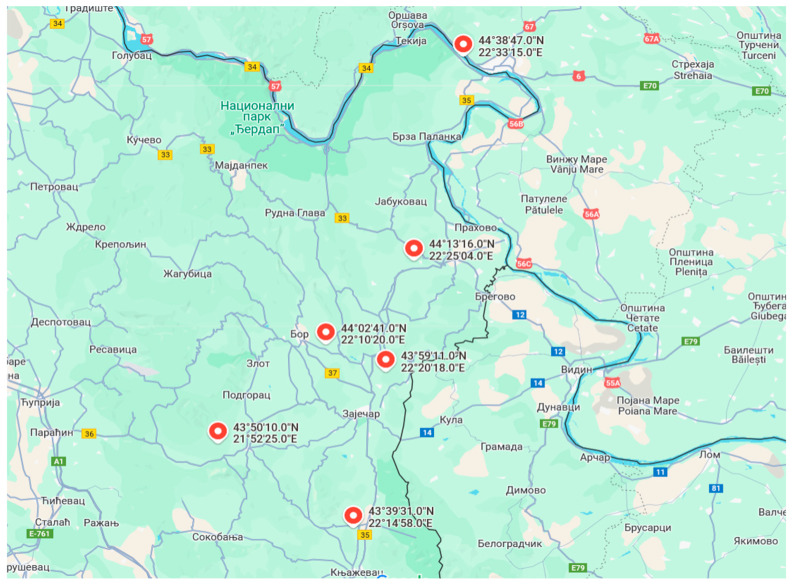
Geographic origin collection of *T. incarnatum* seeds. (Зајечар = Zaječar, Бoр = Bor, Негoтин = Negotin, Кладoвo = Kladovo, Бoљевљац = Boljevac, Књажевац = Knjaževac).

Crimson clover *(Trifolium incarnatum)* seeds were collected from six localities (hereafter referred to as lots) in eastern Serbia, in their mature state (dark pods). The seeds were stored in paper bags under storage conditions (at 10 °C and 50% air humidity) in 2021, 2022, and 2023 (Table 6). Seeds from all localities (Figure 6, Figure 7 and Figure 8) were used in the experiments, with each treatment performed in triplicate.

### 4.1. Water and Air Treatments

Water treatments. The seeds were placed in a wire mesh immersed in water inside a metal kettle with the temperature regulated by a thermostat. Seeds were immersed in hot distilled water at 100 °C for varying durations: 2 min (V1), 4 min (V2), 6 min (V3), 8 min (V4), 10 min (V5), 20 min (V6), 30 min (V7), 45 min (V8), and 60 min (V9). After treatment, a water absorption test was conducted, and seed weight was measured. The seeds were then stored for five days at 5 °C, according to ISTA regulations [58]. Following measurements, the seeds were placed in an incubator, and seed quality parameters were investigated after 6, 24, 30, 48, and 72 h. The control was included in the study without any treatment applied.

Water absorption test. To determine whether the seeds were in a state of physical dormancy, water absorption tests were performed according to the following formula: %Ws = [(Wh − Wi)/Wi] × 100 [59], where Ws is the ratio of relative seed weight multiplied by water uptake, Wh is the weight of the seeds after adding water, and Wi is the initial weight of the seeds in their dry state.

After drying, the seeds were manually separated from the admixture. For all three years, during the autumn sowing period, the following experiments were performed:

Air temperature treatments. Seeds were exposed to different air temperature treatments, such as heating temperatures and alternating heating and cooling temperatures.

- Seeds were heated with different air temperatures and alternating time as follows: T 40 °C for 5 min (T1) and 10 min (T2), T 60 °C for 5 min (T3) and 10 min (T4), T 80 °C for 5 min (T5) and 10 min (T6), T 100 °C for 5 min (T7) and 10 min (T8).
- Seeds were exposed to alternating heating and cooling temperatures (Mix) and various time durations i.e., T40 °C for 5 min and T-20 °C for 10 min (Mix 1), T 40 °C for 10 min and T-20 °C for 10 min (Mix 2), T 60 °C for 5 min and T-20 °C for 10 min (Mix 3), T 60 °C for 10 min and T-20 °C for 10 min (Mix 4), T 80 °C for 20 min and T-20 °C for 20 min (Mix 5), T 100 °C for 30 min and T-20 °C for 30 min (Mix 6).

A water absorption test was performed after the treatments, during which seed weight was measured. The seeds were then stored at 5 °C for five days. After measurements, the seeds were placed in an incubator, and seed quality parameters were assessed after 6, 24, 30, 48, and 72 h.

Seed quality in laboratory tests. Seed quality assessment included: germinated seeds (first and final measurements/counts (GS1) and (GS2)), dormant seeds DoS, dead seeds DeS, and abnormal seedlings AS, expressed as percentages according to the formula: 100% = GS2 + DoS + DeS + AS. The first and final counts were performed on the 4th day (GS1) and on the 7th day (GS2) respectively, after storing the seeds in an incubator in the dark at a temperature of 20 °C according to ISTA regulations [58]. If it was not clear whether the seeds were dead or not, the tetrazolium test was used [60].

Seedling growth. The stem and root lengths in an incubator (germination cabinet) were determined on the same day as final germination (GS2)—on the 7th day, according to the method previously described [36,61].

Field trials. For each lot, 3 × 100 seeds per treatment were counted for each treatment and sown in soil to test seed quality and seedling growth.

In this experiment, seeds were subjected to the same water and air treatments as in previous experiments before being sown in optimally prepared soil. The seeds were hand-sewn at a depth of approximately 0.5 cm in 20 cm × 20 cm base plots. The soil was watered regularly.

Sowing was carried out in the second half of March and the first half of April under mild continental climate conditions (https://www.hidmet.gov.rs/ accessed on 30 March 2023.

The agrochemical properties of the soil used in the experiment were as follows:

Localities;

Bor pH 4.2%, humus 1.48%, P_2_O_5_ 7.06%, K_2_O 9.65%, N 0.14%,

Zaječar pH 3.91%, humus 1.55%, P_2_O_5_ 8.12%, K_2_O 7.05%, N 0.10%,

Boljevac pH 4.77%, humus 2.18%, P_2_O_5_ 6.16%, K_2_O 5.78%, N 0.27%,

Negotin pH 3.03%, humus 1.75%, P_2_O_5_ 6.55%, K_2_O 4.92%, N 0.11%,

Knjaževac pH 4.99%, humus 2.34%, P_2_O_5_ 5.78%, K_2_O 3.95%, N 0.12%,

Kladovo pH 3.12%, humus 1.78%, P_2_O_5_ 3.69%, K_2_O 3.65%, N 0.13%.

The field experiments were carried out near the collection sites.

### 4.2. Statistical Analysis

For statistical analysis of the effect of factors, the analysis of variance (ANOVA-F test) was used for two and three factors. Arcsine transformation was applied to the data. Standard error of the mean (SEM) and coefficient of variation (CV%) were calculated to represent the variability of the data. Tukey’s multiple range test (*p* ≤ 0.05) was used to analyze the effect of treatments using Minitab Inc. Version 16.1.0. State College, PA, USA (https://www.minitab.com/en-us/, accessed on 25 November 2023) [62], and R Core Team. R-Statistics, a Language and Environment for Statistical Computing, R Foundation for Statistical Computing: Vienna, Austria, 2018, free version [63].

## 5. Conclusions

Of the 24 ecologically acceptable treatments investigated in the water treatment group, the longest seed soaking times (V9 and V8) led to the greatest increase in seed weight, indicating seed coat permeability. However, these treatments also damaged the embryo, as confirmed by the increased percentage of dead seeds, leading to a decline in germination.

The optimal treatments involved hot water (V7 and V6), which increased water absorption and reduced the percentage of dormant seeds. The same treatments also decreased the percentage of dead seeds and abnormal seedlings, resulting in germination rates that were 26% and 21% higher than the control. Additionally, seedling stem growth increased by 48% and 34%, while the root growth improved by 36% and 34%, respectively, compared to the control. The series of air temperature treatments generally caused a decrease in water absorption and increased seed dormancy compared to the optimal water treatments. In the combined high- and low-temperature treatment group (Mix), treatments at 80 °C for 20 min and −20 °C for 20 min increased water uptake and reduced dormancy after 24 h in the incubator. These findings highlight new biotechnological approaches in seed quality enhancement of *T. incarnatum* in order to achieve optimal yields in pasture soils.

In the future, the study should be extended to other areas of Serbia, where the use of new agricultural practices improves seed yield and the production potential of pastures.

## Figures and Tables

**Table 1:** Results of the ANOVA-F test for lots, years, and their interactions on the weight of 1000 seeds, along with the standard error of the mean (SEM) and the coefficient of variation (CV%).

Factors	df	1000 Seed Weight	SEM	CV %
Lots (A)	5	ns	0.118	2.92
Years (B)	2	ns	0.125	3.13
A × B	10	ns		

F test, ns—not significant, *p* ≥ 0.05.

**Table 2:** Results of the ANOVA-F test for the water absorption test in relation to treatments, lots, years, and their interactions, along with the standard error of the mean (SEM) and the coefficient of variation (CV%).

Factors	df	Weight of 1000 Seeds	SEM	CV %
Treatments (A)	23	***	5.13	85.3
Lots (B)	5	ns	0.25	4.25
Years (C)	2	ns	0.29	3.93
A × B	115	ns		
A × C	46	ns		
B × C	10	ns		

F test, statistical significance levels: *** *p* ≤ 0.001 ns—not significant *p* ≥ 0.05.

**Table 3:** Results of the ANOVA-F test on seed quality: germination GS, dormant seeds DoS, dead seeds DeS, abnormal seedlings AS, in relation to treatments, lots, years, and their interactions, along with the standard error of the mean (SEM) and the coefficient of variation (CV%).

Factors	df	Seeds (Laboratory Conditions)															Field Conditions		
		GS1	SEM	CV %	GS2	SEM	CV %	DoS	SEM	CV %	DeS	SEM	CV %	AS	SEM	CV %			
																	GS	SEM	CV %
Treatments (A)	23	***	0.829	18.6	**	0.201	20.1	***	1.784	33.4	***	1.404	81.2	*	0.122	52.9	**	0.189	18.9
Lot (B)	5	ns	0.156	4.11	ns	0.163	4.24	ns	0.112	4.31	ns	0.065	15.1	ns	0.082	14.2	ns	0.155	4.11
Year (C)	2	ns	0.143	3.98	ns	0.152	4.14	ns	0.143	4.03	ns	0.071	17.3	ns	0.089	13.9	ns	0.144	4.02
A × B	115	ns			ns			ns			ns			ns			ns		
A × C	46	ns			ns			ns			ns			ns			ns		
B × C	10	ns			ns			ns			ns			ns			ns		

F test, statistical significance levels: * *p* ≤ 0.05, ** *p* ≤ 0.01, ***p ≤ 0.001, ns—not significant (*p* ≥ 0.05), df—degrees of freedom.

**Table 4:** Comparison of treatment means regarding stem and root growth after applied treatments.

Treatments	Stem (cm)	Root (cm)
V1 Water 100 °C 2 min	2.21 d	1.78 e–g
V2 Water 100 °C 4 min	2.29 d	1.82 e–g
V3 Water 100 °C 6 min	2.35 cd	1.85 e–g
V4 Water 100 °C 8 min	2.44 cd	1.88 c–g
V5 Water 100 °C 10 min	2.48 cd	1.91 c–g
V6 Water 100 °C 20 min	3.18 a	2.31 ab
V7 Water 100 °C 30 min	3.25 a	2.34 a
V8 Water 100 °C 45 min	2.89 ab	2.09 a–c
V9 Water 100 °C 60 min	2.78 bc	2.02 c–g
T1 40 °C 5 min	2.48 cd	2.07 a–c
T2 40 °C 10 min	2.51 cd	2.08 a–c
T3 60 °C 5 min	2.46 cd	2.06 a–c
T4 60 °C 10 min	2.36 cd	1.93 c–g
T5 80 °C 5 min	2.32 d	1.92 c–g
T6 80 °C 10 min	2.27 d	1.84 e–g
T7 100 °C 5 min	2.24 d	1.79 e–g
T8 100 °C 10 min	2.18 d	1.77 e–g
Mix1 T 40 °C 5 min + T-20 °C 10 min	2.44 cd	1.99 c–g
Mix2 T 40 °C 10 min +T-20 °C 10 min	2.39 cd	1.82 e–g
Mix3 T 60 °C 5 min + T-20 °C 10 min	2.41 cd	1.81 e–g
Mix4 T 60 °C 10 min + T-20 °C 10 min	2.45 cd	1.89 c–g
Mix5 T 80 °C 20 min +T-20 °C 20 min	2.47 cd	1.88 c–g
Mix6 T100 °C 30 min + T-20 °C 20 min	2.26 d	1.77 e–g
Control treatment	2.19 d	1.72 g
SEM	0.035	0.021
CV%	11.50	8.41

Tukey’s multiple range test, numbers followed by different letters are statistically different at *p* ≤ 0.05.

**Table 5:** Comparison of treatment means regarding stem and root growth after applied treatments under field conditions.

Treatments	Stem (cm)	Root (cm)
+V1 Water 100 °C 2 min	2.28 d	1.81 f–h
V2 Water 100 °C 4 min	2.33 d	1.85 c–h
V3 Water 100 °C 6 min	2.37 d	1.88 c–h
V4 Water 100 °C 8 min	2.49 cd	1.91 c–h
V5 Water 100 °C 10 min	2.52 cd	1.94 c–h
V6 Water 100 °C 20 min	3.26 a	2.35 ab
V7 Water 100 °C 30 min	3.29 a	2.39 a
V8 Water 100 °C 45 min	2.95 ab	2.13 a–c
V9 Water 100 °C 60 min	2.83 bc	2.04 c–g
T1 40 °C 5 min	2.56 cd	2.09 b–e
T2 40 °C 10 min	2.56 cd	2.11 b–d
T3 60 °C 5 min	2.55 cd	2.09 b–e
T4 60 °C 10 min	2.39 d	1.96 c–h
T5 80 °C 5 min	2.36 d	1.97 c–h
T6 80 °C 10 min	2.33 d	1.88 c–h
T7 100 °C 5 min	2.29 d	1.83 d–h
T8 100 °C 10 min	2.24 d	1.81 e–h
Mix1 T 40 °C 5 min + T-20 °C 10 min	2.49 cd	1.87 c–h
Mix2 T 40 °C 10 min +T-20 °C 10 min	2.43 d	1.86 c–h
Mix3 T 60 °C 5 min + T-20 °C 10 min	2.41 d	1.86 c–h
Mix4 T 60 °C 10 min + T-20 °C 10 min	2.48 cd	1.91 c–h
Mix5 T 80 °C 20 min +T-20 °C 20 min	2.51 cd	1.92 c–h
Mix6 T100 °C 30 min + T-20 °C 20 min	2.31 d	1.79 gh
Control treatment	2.24 d	1.75 h
SEM	0.034	0.020
CV%	11.59	8.24

Tukey’s multiple range test, numbers followed by different letters are statistically different at *p* ≤ 0.05.

**Table 6:** Collection time of *T. incarnatum* seeds.

Localities (Lots)	Collection Time Year		
	2021	2022	2023
Bor I	27 May	21 May	25 May
Zaječar II	30 May	29 May	27 May
Boljevac III	2 June	5 June	2 June
Negotin IV	26 May	29 May	29 May
Knjaževac V	28 May	1 June	1 June
Kladovo VI	2 June	29 May	28 June

## Data Availability

All relevant data are included in the manuscript. All other data will be made available by the authors on reasonable request.
